# Prevalence of stunting and associated factors among under-five children in sub-Saharan Africa: Multilevel ordinal logistic regression analysis modeling

**DOI:** 10.1371/journal.pone.0299310

**Published:** 2024-06-13

**Authors:** Wullo Sisay Seretew, Getayeneh Antehunegn Tesema, Bantie Getnet Yirsaw, Girum Shibeshi Argaw

**Affiliations:** 1 Department of Epidemiology and Biostatistics, Institute of Public Health, College of Medicine and Health Sciences and Comprehensive Specialized Hospital, University of Gondar, Gondar, Ethiopia; 2 Department of Nursing, College of Medicine and Health Science, Jigjiga University, Jigjiga, Ethiopia; Debre Tabor University, ETHIOPIA

## Abstract

**Introduction:**

Stunting is still a major public health problem all over the world, it affecting more than one-third of under-five children in the world that leads to growth retardation, life-threatening complication and accelerate mortality and morbidity. The evidence is scarce on prevalence and associated factors of stunting among under-five children in Sub-Saharan Africa for incorporated intervention. Therefore this study aimed to investigate the prevalence and determinants of stunting among under-five children in Sub-Saharan Africa using recent demographic and health surveys of each country.

**Methods:**

This study was based on the most recent Demographic and Health Survey data of 36 sub-Saharan African countries. A total of 203,852(weighted sample) under-five children were included in the analysis. The multi-level ordinal logistic regression was fitted to identify determinants of stunting. Parallel line (proportional odds) assumption was cheeked by Brant test and it is satisfied (p-value = 0.68) which is greater than 0.05. Due to the nested nature of the dataset deviance was used model comparison rather than AIC and BIC. Finally the adjusted odds ratio (AOR) with 95% CI was reported identify statistical significant determinants of stunting among under-five children.

**Results:**

In this study, the prevalence of stunting among under-five children in Sub-Saharan Africa 34.04% (95% CI: 33.83%, 34.24%) with a large difference between specific countries which ranges from 16.14% in Gabon to 56.17% in Burundi. In the multi-level ordinal logistic regression good maternal education, born from mothers aged above 35 years, high household wealth status, small family size, being female child, being female household head, having media exposure and having consecutive ANC visit were significantly associated with lower odds of stunting. Whereas, living from rural residence, being 24–59 month children age, single or divorced marital status, higher birth order and having diarrhea in the last two weeks were significantly associated with higher odds of stunting.

**Conclusion:**

Stunting among under-five children is still public health problem in Sub-Saharan Africa. Therefore designing interventions to address diarrhea and other infectious disease, improving the literacy level of the area and increase the economic level of the family to reduce the prevalence of stunting in the study area.

## Background

Stunting is defined as a height that is more than two standard deviations below the World Health Organization (WHO) child growth standard media. Stunting is still a major public health problem all over the world [1, 2]. World Health organization and the World Bank indicate that the number of stunted children is approximately 150 million, accounting 22% of the children in the world [3]. Furthermore the number of stunting children is concentrated in low-middle- income countries (47%) compared to high-income countries (10%). Around 58.7 million stunted children live in Africa [4], among this majority of the stunted children found in Sub-Sahara Africa regions. Nearly, half of infant and child mortalities in Sub-Sahara Africa are associated with stunting, which culminate in 9% decline in the region’s workforce, and thus, hampering the economic growth of the nation [5–7].

Globally, about 144 million under-five children are suffering from stunting, of which 92% are in Low and lower-middle-income countries and Sub-Saharan countries share just over a third of the burden [7]. Considering the adverse effects of malnutrition, particularly on the most vulnerable groups, such as children below 5 years of age, each of the Sub-Saharan Africa countries government initiated and implemented a range of nutritional programs and policies to effectively reduce child malnutrition, whose core targets include a substantial reduction of childhood under-nutrition [6]. Despite all these efforts, there has been little or no reduction in stunting among children below 5 years of age in Sub-Saharan Africa. Therefore, designing effective nutritional intervention policies targeting children at heightened odds of chronic under nutrition is crucial and entails a clear delineation of factors associated with stunted children in the region. Stunting can be caused by various factors such as parental, socio-demographic, and economic status, as well as cultural practices and environmental and other health related variables. For instance, poverty, low parental education, lack of sanitation, low food intake, poor feeding practices, inadequate breastfeeding, repeated infections, family size and birth interval are regarded as key determinants of stunting [8, 9].

To the best of our searching, there is limited evidence regarding stunting prevalence and associated factors among under-five children in the study area in order to improve child nutrition in Sub-Saharan Africa, an individual level and community level based study with sufficient sample size is needed to provide a comprehensive understanding of the factors associated with level of stunting. Hence, the main aim of this study is to utilize data from 36 Sub-Saharan Africa (12 East Africa country, 13 West Africa country, 7 Central Africa and 4 South Africa country) Demographic and Health Survey to determine the factors that are statistical significantly associated with stunting among under-five children after controlling for potential confounding factors. Findings from this study can be useful to policy makers, program developers, global health sector higher officials and public health researchers in formulating effective interventions and to reach a deliberate equity-driven policy targeting interventions for the most vulnerable population as early as possible.

## Methods

### Study design and setting

Data analysis was done based on the most recent Demographic Health Survey datasets conducted in 36 sub-Saharan African countries. DHS is a nationally representative survey that provides data for monitoring indicators of nutrition and health. The authorization to use the data was approved from the measure DHS program.

### Study population and sampling procedure

All under-five children in Sub-Saharan Africa within the five years preceding the recent survey of each Sub-Saharan Africa countries were the source population and All under-five children in Sub-Saharan Africa who were in the selected clusters were considered as the study population, Children in the study who were in the selected clusters that complete data all information of the interesting variable were included. Children with incomplete information of the interesting variables were excluded from the analysis. The nature of DHS, some of the regions or counties were oversampled and some were under sampled in particular in large counties or regions. The data were weighted before any statistical analysis to restore the representativeness of the data and to get a reliable estimate and standard error. A total weighted sample of 203,852 under five children was including. A multistage cluster sampling technique was employed to recruit the samples using Enumeration areas as primary sampling units and households as secondary sampling units.

### Variables of the study

The outcome variable for our study was the stunting status of children, which was an ordered category variables categorized in to three ordinal categories. Those categories was normal, moderate stunting (height-for-age Z-score (HAZ) <−2SD) and sever stunting (height-for-age Z-score (HAZ) <−3SD).

The explanatory variables were classified in to individual-and community-level variables. Type of residence, Child age, child sex, household wealth, maternal education, maternal age, sex of household head, child twin, birth order, media exposure, number of ANC visits, place of delivery, husband education, birth size, and preceding birth interval were individual-level variables. Residence and sub-Saharan African region were community-level variable.

### Data management and analysis

STATA version 17 software was used for data management and analysis. Descriptive statistics were done using frequencies and percentages. For ordered nature of the response variable and the hierarchal nature of the DHS data violates the independent assumptions of the standard binary logistic regression model and because of this, we cannot use the standard logistic regression analysis rather than we can fitted a multilevel ordinal logistic regression analysis. They are different types of ordinal logistic regression models. The most common logistic regression models are: Proportional Odds Model, Partial Proportional Odds Model with Restrictions and Partial Proportional Odds Model without Restrictions, Continuous Ratio Model and Stereotype Model. In most studies of the glove use Proportional Odds Model is the most commonly used ordinal logistic regression model. The fundamental assumption in an ordinal logistic regression model is the Parallel line assumption. If the data satisfies the Parallel line assumption, the proportional odds model can be used.

We have checked the parallel line assumptions, which state that the effects of all independent variables are constant across categories of the response variable. The Brant test discovered that the parallel line assumption was fulfilled (p>0.05). In addition, the DHS data has a hierarchical nature. For that reason, children were nested within a cluster, and we assume that study subjects in the same cluster may distribute similar characteristics to participants in another cluster. This implies the want to take into account the heterogeneity between clusters by using an advanced model. Then, a multilevel proportional odds model was performed. As a result, because the Brant test was met, the multilevel proportional odds model gave a single Odds Ratio (OR) for an explanatory variable (severe vs. moderate/ normal, and severe/moderate vs. normal).

Whereas conducting a multilevel ordinal logistic regression analysis four models; the null model containing only the response variable, model I and II containing individual and community level variables, respectively and model III, which contains both individual and community level variables were fitted. Interclass Correlation Coefficient (ICC), Median Odds Ratio (MOR) and Percentage Change in Variance (PCV) were checked to assess the clustering effect. Since these models were nested, we used deviance to check the model comparison and the model with the lowest deviance were chosen as the best model for the data. Both bi-variable and multivariable multilevel ordinal logistic regression was done and variables with a p-value of <0.2 in the bi-variable multilevel ordinal logistics analysis were considered for multivariable multilevel ordinal logistics analysis. Finally, variables with P-value <0.05 in the multivariable multilevel ordinal logistics analysis were identified as significant factors associated with stunting.

### Ethical approval and consent to participate

Ethical approval for this study was not necessary since this study used existing public domain survey data sets. We requested the data from the MEASURE DHS Program and permission was granted to download and use the data for this study. There are no names of individuals or household addresses in the data files.

## Results

### Socio-demographic characteristic of study participants

The prevalence of stunting among under-five children in Sub-Saharan Africa 34.04% (95% CI: 33.83%, 34.24%) with a large difference between specific countries which ranges from 16.14% in Gabon to 56.17% in Burundi Table 1.

**Table 1:** Number of study participants, prevalence of stunting and year of the survey for each country of our study.

Sub-Saharan Africa region		Sample Size (n = 203 852)	Prevalence of stunting	Year of the survey
Central Africa	Angola	5905	37.42	2015/2016
	Chad	9805	39.75	2014/2015
	Congo	3859	23.45	2011/2012
	DR. Congo	7900	42.56	2013/2014
	Cameron	5053	32.32	2011
	Gabon	2792	16.14	2012
	São Tomé’s Príncipe	1284	29.63	2008/2009
East Africa	Burundi	6222	56.17	2016/2017
	Comoros	2468	29.73	2018
	Ethiopia	9588	38.70	2016
	Kenya	17 291	23.01	2014
	Madagascar	4994	50.55	2021
	Malawi	5159	36.82	2015/2016
	Mozambique	9895	43.16	2011
	Rwanda	3593	38.13	2019/2020
	Tanzania	8814	34.45	2015/2016
	Uganda	4350	28.38	2016
	Zambia	8612	34.69	2018
	Zimbabwe	5212	26.65	2013/2014
**South Africa**	Lesotho	1298	32.85	2014
	Namibia	1438	22.33	2013
	Swaziland	2070	28.00	2006/2007
	South Africa	1080	26.55	2016
**West Africa**	Burkina Faso	6660	34.67	2010
	Benin	11 717	31.84	2017/2018
	Coˆte d’Ivoire	3041	29.97	2011
	Ghana	2636	18.20	2014
	Gambia	2939	24.33	2019/2020
	Guinea	3374	31.41	2018
	Mali	8858	26.94	2018
	Nigeria	11 407	36.70	2018
	Niger	5113	43.54	2012
	Liberia	2754	30.36	2019/2020
	Sierra leone	4141	37.98	2019
	Senegal	3357	26.64	2010/2011
	Togo	9173	26.95	2013/2014

Among a total of 203,852 study participants, of this study participants approximately one-thirds (38.96%) of the mothers did not have formal education. Nearly half of (53.34%) of the children’s mothers were aged 25–35 years. Around two-third of the (64.06%) children’s mothers were at list one ANC follow up during her pregnancy time. Most of the study participants (96.9%) were single birth types. Regarding place of delivery around two-thirds of (66.39%) the children got birth at a health facility and 56.76% participants had 1–3 birth order history. About 69.42% of the study participants lived in rural areas.

We looking the wealth status of the study participants 35.67%, 20.08%, 44.25% were rich, middle and poor respectively. About 170,892 (83.87%) had no diarrhea and fever in the last two weeks. Male (50.32%) and female (49.68%) children were almost equally represented. The majority of the study participants (72.75%) were married marital status. Around three-fourth of (79.36%) the household heads were males, and the majority (94.92%) of the study participants were Delivery by without caesarean section Table 2.

**Table 2:** Descriptive characteristics of the study participants in Sub-Saharan Africa.

Variable	Weighted Frequency(N)	Weighted frequency (%)
**Maternal education level**		
No education	79432	38.96
Primary	72871	35.75
Secondary and above	51549	25.29
**Maternal age**		
Below 25	56907	27.92
25–35	108739	53.34
Above 35	38206	18.74
**Birth type**		
Single	197 541	96.90
Twin	6311	3.10
**Birth order**		
1–3	115707	56.76
4–6	61740	30.29
>6	26405	12.95
**Place of delivery**		
Health facility	135342	66.39
Home	68510	33.61
**ANC visit**		
No	73271	35.94
Yes	130581	64.06
**Place of residence**		
Urban	62329	30.58
Rural	141523	69.42
**Sex of household head**		
Male	161784	79.36
Female	42068	20.64
**Wealth status**		
Poor	90211	44.25
Middle	40922	20.08
Rich	72719	35.67
**Family size**		
<6	83837	41.12
6–9	85816	42.10
>9	34199	16.78
**Source of drink water**		
Protected	107747	52.86
Unprotected	96076	47.14
**Marital status**		
Married	148295	72.75
Single/divorced/separated	55557	27.25
**Sex of a child**		
Male	102582	50.32
Female	101270	49.68
**Child`s age**		
<6 months	25640	12.58
6–23 months	61744	30.29
24–59 months	116468	57.13
**History of diarrhea two weeks prior to the survey**		
No	170892	83.87
Yes	32867	16.13
**History of intake of parasite drugs for mothers during pregnancy**		
Yes	62236	45.82
No	73586	54.18
**Work status**		
Yes	120635	59.18
No	83217	40.82
**Media exposure**		
Yes	134568	66.01
No	69284	33.99
**Husband education level**		
No education	61109	34.16
Primary	101901	56.96
Secondary and above	15893	8.88
**Delivery by caesarean section**		
Yes	10276	5.08
No	192190	94.92
**Size of child at birth**		
Small	68740	35.30
Normal	92415	47.46
Large	33581	17.24

### Prevalence of stunting in sub-Saharan Africa

The overall prevalence of stunting among under-five children was 34.04% [95% CI: 33.83%, 34.24%]. Our study showed that 20.13% [95% CI: 19.95%, 20.30%] of under-five children had moderate stunting and 13.91% [95% CI: 13.76%, 14.06%] severe stunting. The highest prevalence of stunting was found in children whose mothers were not ANC visit, place of delivery at home and poor wealth status which was 41.32%, 40.76% and 40.54%, respectively. Regarding the severity of stunting, the highest prevalence of severe stunting was found in children whose family had no media exposure (19.91%) and children got from illiterate fathers (18.45%) and the highest prevalence of moderate stunting were found small size of child at birth (23.20%) and the age of the child 24–59 months(22.66%) Table 3.

**Table 3:** The prevalence of stunting based on the individual level, community level, child and maternal characteristics in sub-Saharan Africa.

Characteristics	Categories	Stunting status			Over all stunting pr for each categories
		Sever	Moderate	Normal	
Maternal education level	No educated	18.32	21.49	60.19	39.81
	Primary	13.98	21.87	64.15	35.85
	Secondary and above	7.02	15.56	77.42	22.58
Maternal age	Below 25	13.94	20.97	65.09	34.91
	Between 25& 35	13.70	19.60	66.70	33.30
	Above 35	14.46	20.34	65.20	34.80
Birth type	Single	13.64	19.93	66.43	33.57
	Twin	22.43	20.13	65.96	34.04
Birth order	1–3	12.54	19.53	67.93	32.07
	4–6	15.07	20.73	64.19	35.81
	>6	17.19	21.31	61.51	38.49
Place of delivery	Health facility	11.43	19.21	69.36	30.64
	Home	18.80	21.94	59.26	40.76
ANC visit	Yes	11.57	18.94	69.49	30.51
	No	18.07	22.24	59.68	41.32
Place of residence	Urban	8.50	16.20	75.30	24.70
	Rural	13.91	20.13	65.96	34.04
Sex of household head	Male	14.15	20.09	65.76	34.24
	Female	13.00	20.27	66.73	33.27
Wealth status	Poor	17.80	22.74	59.46	40.54
	Middle	13.97	21.28	64.75	35.25
	Rich	9.05	16.23	74.72	25.28
Family size	<6	12.90	19.97	67.13	32.87
	6–9	14.54	20.39	65.07	34.93
	>9	14.81	19.85	65.34	34.66
Source of drink water	Protected	12.08	18.89	69.03	30.97
	Unprotected	15.96	21.52	62.52	37.48
Marital status	Married	14.18	20.10	65.72	34.28
	Single/divorced/separated/widowed	13.19	20.20	66.61	33.39
Sex of a child	Male	15.38	21.00	63.62	36.38
	Female	12.42	19.23	68.34	31.66
Child`s age	<6 months	6.13	9.77	84.11	15.89
	6–23 months	12.57	19.64	67.79	32.21
	24–59 months	16.33	22.66	61.01	38.99
History of diarrhea two weeks prior to the survey	Yes	13.55	19.95	66.50	33.50
	No	15.77	21.06	63.17	36.83
History of intake of parasite drugs for mothers during pregnancy	Yes	11.23	18.89	69.88	30.12
	No	14.19	19.48	66.33	33.67
Work status	Yes	14.08	20.66	65.26	34.74
	No	13.66	19.35	66.99	33.01
Media exposure	Yes	11.33	18.90	69.77	30.23
	No	18.91	22.50	58.58	41.42
Husband education level	No education	18.45	21.38	60.17	39.87
	Primary	15.24	22.19	62.57	37.43
	Secondary & above	9.73	17.20	73.07	26.93
Delivery by caesarean section	Yes	8.85	15.86	75.29	24.71
	No	14.14	20.36	65.50	34.50
Size of child at birth	Small	18.80	23.20	58.00	42.00
	Normal	13.74	20.66	65.60	34.40
	Large	12.52	18.19	69.29	30.71

### Determinants of stunting among under-five children

To identify the determinants of stunting, the bi-variable analysis was conducted and a variable p-value <0.02 includes in the multivariable analysis. Both the individual and country-level factors were included simultaneously, sex of household head, sex of a child, media exposure, ANC visit, place of delivery, birth order, maternal education level, wealth status of the family, source of drink water, father education level, size of a child at birth, age of the child, maternal age and place of residence were statistically significant with stunting.

Children who were the 4^th^-6^th^ and greater than 6^th^ birth order were 1.11 times (AOR = 1.11, 95% CI: 1.07, 1.15) and 1.23 times (AOR = 1.23, 95% CI: 1.17, 1.29) higher odds of stunting compared to 3^rd^ and below birth order respectively. Female headed households had 0.97(AOR = 0.97, 95%CI: 0.94–0.99) times lower odds of stunting compared with male headed households.

Looking at educational status of mother, mother with primary (AOR = 0.95, 95%CI: 0.92–0.98) and secondary and above educational status (AOR = 0.70, 95%CI: 0.67–0.73) had lower odds of stunting compared with illiterate mother. The odds of being at higher stunting among children who took drugs for the intestinal parasite in the last six months were decreased by 3% (AOR = 0.97, 95% CI: 0.95, 0.99) than those who did not take drugs. Children who had diarrhea in the last two weeks had 1.11times (AOR = 1.11, 95% CI: 1.08, 1.15) higher odds of a higher level of stunting compared to children who did not have diarrhea Table 4.

**Table 4:** Multivariate ordinal logistic regression analysis of stunting against individual level and community level characteristics.

Characteristics	Null model	Model I	Model II	Model III
		AOR with 95%CI	AOR with 95%CI	AOR with 95%CI
Sex of household head				
female			0.96(0.93, 0.99)	0.97(0.94, 0.99)*
Sex of a child				
Female			0.75(0.73, 0.76)	0.75(0.73, 0.76)*
Media exposure				
yes			0.81(0.79, 0.83)	0.82(0.80, 0.84)*
ANC Visit				
Yes			0.81(0.77, 0.84)	0.80(0.77, 0.84)*
Place delivery				
Home			1.13(1.10, 1.17)	1.12(1.09, 1.15)*
Birth order				
4–6 order			1.11(1.07, 1.15)	1.11(1.07, 1.15)*
>6 order			1.24(1.18, 1.3)	1.23(1.17, 1.29)*
Twin status				
Twin			2.09(1.93, 2.28)	2.10(1.93, 2.28)*
Maternal educationlevel				
Primary			0.95(0.92, 0.98)	0.95(0.92, 0.98)*
Secondary and above			0.68(0.66, 0.71)	0.70(0.67, 0.73)*
Wealth status				
Middle			0.93(0.90, 0.97)	0.95(0.92, 0.98)*
Rich			0.76(0.73, 0.78)	0.81(0.79, 0.84)*
Family size				
6–9			0.96(0.93, 0.99)	0.96(0.93, 0.99)*
>9			0.99(0.96, 1.03)	0.99(0.96, 1.03)
Source of drink water				
Protected			0.92(0.90, 0.94)	0.94(0.92, 0.97)*
Marital status				
single/divorced/separated			1.06(1.03, 1.09)	1.07(1.04, 1.10)*
Age of child				
6–23 month			2.75(2.64, 2.87)	2.75(2.64, 2.87)*
24–59 month			4.25(4.07, 4.43)	4.25(4.07, 4.43)*
History of diarrhea two weeks prior to the survey				
Yes			1.11(1.08, 1.15)	1.11(1.08, 1.15)*
History of intake of parasite drugs for mothers during pregnancy				
Yes			0.97(0.94, 0.99)	0.97(0.95, 0.99)*
Husband education level				
Primary			0.82(0.77, 0.88)	0.83(0.78, 0.88)*
Secondary and above			0.72(0.68, 0.76)	0.74(0.70, 0.78)*
Delivery by caesarean section				
Yes			0.90(0.84, 0.96)	0.91(0.85, 0.97)*
Size of a child at birth				
Normal			1.21(1.17, 1.23)	1.20(1.17, 1.23)*
Low			1.70(1.64, 1.76)	1.70(1.64, 1.76)*
Maternal age				
25–35			0.83(0.80, 0.86)	0.83(0.81, 0.86)*
Above 35			0.71(0.68, 0.75)	0.72(0.68, 0.75)*
Residence				
Rural		1.78(1.74, 1.82)		1.21(1.17, 1.25)*
cut1	2.01(1.98, 2.05)	1.11(1.08, 1.13)	1.15(1.08, 1.22)	1.34(1.26, 1.41)
cut2	1.87(1.85, 1.89)	2.29(2.26, 2.31)	2.38(2.31, 2.45)	2.57(2.49, 2.65)
**Random effect result**				
Log-likelihood	-179278.5	-177790.36	-97830.07	-97770.81
Deviance	358,557	355,580.72	195,660.14	195,541.62
ICC	0.09(0.07, 0.11)			
MOR	1.45(1.38, 1.53)			

AOR = Adjusted Odds Ratio: CI: Confidence Interval: ICC = Intra-class Correlation Coefficient: MOR: Median Odds Ratio:

*P-value < 0.05

### Proposed model comparison

A model with low (195,541.62) deviance and high log-likelihood (-97770.82) was the best model. The combined individual level and community level multilevel ordinal logistic regression model (model III) was the best-fitted model in our study.

## Discussion

Our study shows that the extent of stunting among under-five children in Sub-Saharan Africa is high. This might be related to the limited health service infrastructure coverage [4], highly prevalence of infectious disease areas like hookworm, malaria and poor sanitation system of the environment [10, 11] all these factors might be a comfort zone of spread of parasites and their transmissions [12].

The final model of our study indicates that children born to illiterate mother had higher odds of stunting compared to literate mother. It’s consistent with studies conducted in Bolivia [13], Bangladesh [14] and Indonesia [8]. It could be due to education can positively influence feeding practice of their children, good understanding regarding with child health and nutrition, encourage to follow antenatal care services during pregnancy and educated mothers are more likely to utilize child health services, which can have a positive effect on their children’s health outcomes [15].

Sex of a child was highly associated with stunting in this study, evidence show that male children had higher odds of stunting compared to female children. It is in line with studies conducted in Nigeria, Kenya, Tanzania and India [16–19]. This might be due to male children and female children are different with protein and gene expression placenta especially during adverse conditions in addition to this it might be the mi RNA could show down regulation in male and up regulation in female. This makes males have more stunting than of females. Place of delivery was strong association with stunting. Hence, children who delivered at home had higher odds of stunted as compared with children delivered at health facility. This finding is similar studies conducted in Democratic Republic Congo, Uganda and Iran [20–22] this may be occurred due to lack of adequate new born care at home, the baby may not take the immediate nutrition after birth and its difficult to solve complications.

The result of the current studies showed that children taking drugs for the intestine parasite in the last six months had less odds of stunting compared to children had no taking drugs for the intestine parasite in the last six months it is consistent with previous studies reported in Eastern Nepal, Ethiopia and Tanzania, [11, 23, 24] it could be due to the routine vitamin A supplementation, protective or curative deforming combined with Integrated Management of Neonatal and Childhood Illness may benefit to decrease the problem of childhood stunting. The current study showed that the odds of stunting was higher among twin pregnancies mother compared with that of singleton pregnancies mother which is in line with study conducted in South Africa [25], East Africa [26] and Italy [27]. This might be multiple pregnancies can lead preterm labor, low birth weight, delivery complication, high risk of birth infection and biological immaturity. Children from large family size had higher stunted as compared with small family size it is consistent with previous studies reported in Nigeria [28], Iran and Indonesia [29, 30] this may be occurred due to children from large family size may not get sufficient nutrients like vitamin B12 and iron.

The study at hand, children born to mother’s age less than 25 years higher odds of stunting compared to children born to mother age and above. It is similar with studies reported in Tanzania [6, 9, 18, 31]. The possible reasons might be babies born to aged mother are less likely to be preterm and low birth weight as compared to younger mothers. Our study revealed that children from families with poor household wealth had higher odds of stunting compared to children from rich household wealth. It is in line with study finding in India, Brazil and Kenya [32–34] it may due to poverty is strong association with food insecurity and low high income are more likely to purchase nutrient-rich foods and also the children from high income level might get necessary health services during pregnancy and the child might be provided good care than family with low-income levels. Other significant predictor that was associated with stunting was source of drinking water. Those children whose families use drinking water from protected source were less likely to be stunted as compared to those who used unprotected source. This finding similar with studies conducted in Indonesia, Malawi and Tanzania [11, 12, 35, 36]. This might be because of unsafe water aggravates the spread of water-borne diseases that can affect the health status of children.

Exposure to diarrheal diseases two weeks prior to the survey had significant association with stunting. The finding of this study was in line with the other studies conducted in different parts of the world like USA, Virginia and Peru [37–40]. All the referenced studies confirmed that exposure to diarrhea two weeks prior to the survey had association with stunting. This might be because of loss of fluids and electrolytes, loss of food appetite and absorption in the intestine.

### Strength and limitations of the study

This study has several strengths. First, the study was based on important weighted nationally representative DHS surveys of 36 Sub-Saharan African countries that were to get a reliable estimate. Secondly, multilevel ordinal logistic regression was fitted by considering the hierarchical nature of the DHS data to get identify community and individual-level predictors of stunting among under-five children. Furthermore, the study was based on the large sample size; this could increase the power of the study to get the true effect of the predictors. This finding should be interpreted in light of the following limitations. This study was based upon recall by mothers and it is prone to recall bias and the DHS survey year was not the same in all countries, it was based on DHS conducted 2008 to 2019. This might overestimate or underestimate the prevalence of stunting among under-five children.

## Conclusion and recommendation

The prevalence of stunting among under-five children in Sub-Saharan Africa was high. Our findings indicate that stunting was a major public health problem in Sub-Saharan Africa. Maternal education level, maternal age, birth order, wealth status of family, family size, child age, sex of household head, husband education level, source of drink water, taking drugs for an intestinal parasite, diarrhea in the last two weeks, family size was found statistical significant parameters of stunting in Sub-Saharan Africa region. So, health sector policy makers should focus on improving the education access of the area, give intervention to address diarrhea and other infectious disease, increase the economic level of the family, improving sanitation and water access of the community to reduced the burden of stunting among under-five children for the betterment of the continuity of the generations.

## Supporting information

S1 Data (DTA)

## Abbreviations

ANC: Antenatal Care
AOR: Adjusted Odds Ratio
CSA, CI: Confidence Interval
DHS: Demographic and Health Survey
EAs: Enumeration Areas
HAZ: Z-score for Height for-Age
ICC: Intra-cluster Correlation Coefficient
LLR: Log likelihood ratio
SSA: Sub-Saharan Africa
WHO: World Health Organization

## References

[1] Organization WH. Childhood stunting: challenges and opportunities: report of a webcast colloquium on the operational issues around setting and implementing national stunting reduction agendas, 14 October 2013-WHO Geneva. 2014.

[2] Kraay A. Methodology for a World Bank human capital index. World Bank Policy Research Working Paper. 2018(8593).

[3] Mapping child growth failure across low-and middle-income countries. Nature. 2020;577(7789):231–4. doi: 10.1038/s41586-019-1878-8 31915393 PMC7015855

[4] Pelletier DL, Frongillo EA Jr, Schroeder DG, Habicht J-P. The effects of malnutrition on child mortality in developing countries. Bulletin of the World Health Organization. 1995;73(4):443. 7554015 PMC2486780

[5] Konje JC, Ladipo OA. Nutrition and obstructed labor. The American journal of clinical nutrition. 2000;72(1):291S–7S. doi: 10.1093/ajcn/72.1.291S 10871595

[6] Akombi BJ, Agho KE, Merom D, Renzaho AM, Hall JJ. Child malnutrition in sub-Saharan Africa: A meta-analysis of demographic and health surveys (2006–2016). PloS one. 2017;12(5):e0177338. doi: 10.1371/journal.pone.0177338 28494007 PMC5426674

[7] Ghodsi D, Omidvar N, Eini-Zinab H, Rashidian A, Raghfar H. Impact of the national food supplementary program for children on household food security and maternal weight status in Iran. International journal of preventive medicine. 2016;7. 27833722 10.4103/2008-7802.190605 PMC5036276

[8] Agho KE, Inder KJ, Bowe SJ, Jacobs J, Dibley MJ. Prevalence and risk factors for stunting and severe stunting among under-fives in North Maluku province of Indonesia. BMC pediatrics. 2009;9(1):1–10. doi: 10.1186/1471-2431-9-64 19818167 PMC2765943

[9] Aguayo VM, Nair R, Badgaiyan N, Krishna V. Determinants of stunting and poor linear growth in children under 2 years of age in India: An in‐depth analysis of Maharashtra’s comprehensive nutrition survey. Maternal & child nutrition. 2016;12:121–40. doi: 10.1111/mcn.12259 27187911 PMC5084823

[10] Spencer PR, Sanders KA, Judge DS. Growth curves & the international standard: How children’s growth. Journal of Clinical Nutrition. 2007;43:711–22.

[11] Mshida HA, Kassim N, Mpolya E, Kimanya M. Water, sanitation, and hygiene practices associated with nutritional status of under-five children in semi-pastoral communities Tanzania. The American journal of tropical medicine and hygiene. 2018;98(5):1242. doi: 10.4269/ajtmh.17-0399 29532770 PMC5953357

[12] Lelijveld N, Seal A, Wells JC, Kirkby J, Opondo C, Chimwezi E, et al. Chronic disease outcomes after severe acute malnutrition in Malawian children (ChroSAM): a cohort study. The Lancet Global Health. 2016;4(9):e654–e62. doi: 10.1016/S2214-109X(16)30133-4 27470174 PMC4985564

[13] Frost MB, Forste R, Haas DW. Maternal education and child nutritional status in Bolivia: finding the links. Social science & medicine. 2005;60(2):395–407. doi: 10.1016/j.socscimed.2004.05.010 15522494

[14] Rahman MS, Howlader T, Masud MS, Rahman ML. Association of low-birth weight with malnutrition in children under five years in Bangladesh: do mother’s education, socio-economic status, and birth interval matter? PloS one. 2016;11(6):e0157814. doi: 10.1371/journal.pone.0157814 27355682 PMC4927179

[15] Nasim N, El-Zein A, Thomas J. A review of rural and peri-urban sanitation infrastructure in South-East Asia and the Western Pacific: Highlighting regional inequalities and limited data. International Journal of Hygiene and Environmental Health. 2022;244:113992. doi: 10.1016/j.ijheh.2022.113992 35752101

[16] Akombi BJ, Agho KE, Hall JJ, Merom D, Astell-Burt T, Renzaho AM. Stunting and severe stunting among children under-5 years in Nigeria: A multilevel analysis. BMC pediatrics. 2017;17:1–16. 28086835 10.1186/s12887-016-0770-z PMC5237247

[17] Bloss E, Wainaina F, Bailey RC. Prevalence and predictors of underweight, stunting, and wasting among children aged 5 and under in western Kenya. Journal of tropical pediatrics. 2004;50(5):260–70. doi: 10.1093/tropej/50.5.260 15510756

[18] Chirande L, Charwe D, Mbwana H, Victor R, Kimboka S, Issaka AI, et al. Determinants of stunting and severe stunting among under-fives in Tanzania: evidence from the 2010 cross-sectional household survey. BMC pediatrics. 2015;15:1–13. 26489405 10.1186/s12887-015-0482-9 PMC4618754

[19] Fenske N, Burns J, Hothorn T, Rehfuess EA. Understanding child stunting in India: a comprehensive analysis of socio-economic, nutritional and environmental determinants using additive quantile regression. PloS one. 2013;8(11):e78692. doi: 10.1371/journal.pone.0078692 24223839 PMC3817074

[20] Kandala N-B, Madungu TP, Emina JB, Nzita KP, Cappuccio FP. Malnutrition among children under the age of five in the Democratic Republic of Congo (DRC): does geographic location matter? BMC public health. 2011;11:1–15. 21518428 10.1186/1471-2458-11-261 PMC3111378

[21] Kikafunda JK, Walker AF, Collett D, Tumwine JK. Risk factors for early childhood malnutrition in Uganda. Pediatrics. 1998;102(4):e45–e. doi: 10.1542/peds.102.4.e45 9755282

[22] Mansori K, Shadmani FK, Mirzaei H, Azad RV, Khateri S, Hanis SM, et al. Prevalence of stunting in Iranian children under five years of age: Systematic review and meta-analysis. Medical journal of the Islamic Republic of Iran. 2018;32:103. doi: 10.14196/mjiri.32.103 30815398 PMC6387800

[23] Limbu DS, Shrestha S, Bantawa K, Majhi R, Kharel M. Intestinal Parasitic Infections and Associated Risk Factors Among School-going Children of Age 1–5 years in Dharan, Eastern Nepal. Himalayan Journal of Science and Technology. 2021;5(01):26–35.

[24] Amsalu ET, Akalu TY, Gelaye KA. Spatial distribution and determinants of acute respiratory infection among under-five children in Ethiopia: Ethiopian Demographic Health Survey 2016. PloS one. 2019;14(4):e0215572. doi: 10.1371/journal.pone.0215572 31009506 PMC6476529

[25] Tako E, Beebe SE, Reed S, Hart JJ, Glahn RP. Polyphenolic compounds appear to limit the nutritional benefit of biofortified higher iron black bean (Phaseolus vulgarisL.). Nutrition journal. 2014;13(1):1–9. 24669764 10.1186/1475-2891-13-28 PMC3976557

[26] Kruger H. Maternal anthropometry and pregnancy outcomes: a proposal for the monitoring of pregnancy weight gain in outpatient clinics in South Africa. Curationis. 2005;28(4):40–9. doi: 10.4102/curationis.v28i4.1012 16450558

[27] Branca F, Ferrari M. Impact of micronutrient deficiencies on growth: the stunting syndrome. Annals of nutrition and metabolism. 2002;46(Suppl. 1):8–17. doi: 10.1159/000066397 12428076

[28] Ajao K, Ojofeitimi E, Adebayo A, Fatusi A, Afolabi O. Influence of family size, household food security status, and child care practices on the nutritional status of under-five children in Ile-Ife, Nigeria. African journal of reproductive health. 2010;14(4). 21812205

[29] Mahyar A, Ayazi P, Fallahi M, HAJI SJT, Farkhondehmehr B, Javadi A, et al. Prevalence of underweight, stunting and wasting among children in Qazvin, Iran. 2010.

[30] Mahmudiono T, Sumarmi S, Rosenkranz RR. Household dietary diversity and child stunting in East Java, Indonesia. Asia Pacific journal of clinical nutrition. 2017;26(2):317–25. doi: 10.6133/apjcn.012016.01 28244712

[31] Haile D, Nigatu D, Gashaw K, Demelash H. Height for age z score and cognitive function are associated with Academic performance among school children aged 8–11 years old. Archives of Public Health. 2016;74(1):1–7. doi: 10.1186/s13690-016-0129-9 27141306 PMC4852419

[32] Chilton M, Chyatte M, Breaux J. The negative effects of poverty & food insecurity on child development. Indian Journal of Medical Research. 2007;126(4):262–72. 18032801

[33] Fotso J-C, Kuate-Defo B. Socioeconomic inequalities in early childhood malnutrition and morbidity: modification of the household-level effects by the community SES. Health & place. 2005;11(3):205–25. doi: 10.1016/j.healthplace.2004.06.004 15774328

[34] Deshmukh PR, Sinha N, Dongre AR. Social determinants of stunting in rural area of Wardha, Central India. Medical journal armed forces India. 2013;69(3):213–7. 24600112 10.1016/j.mjafi.2012.10.004 PMC3862661

[35] Torlesse H, Cronin AA, Sebayang SK, Nandy R. Determinants of stunting in Indonesian children: evidence from a cross-sectional survey indicate a prominent role for the water, sanitation and hygiene sector in stunting reduction. BMC public health. 2016;16(1):1–11. doi: 10.1186/s12889-016-3339-8 27472935 PMC4966764

[36] WHO E. Water safety planning for small community water supplies: step-by-step risk management guidance for drinking-water supplies in small communities. Geneva: World Health Organization. 2012.

[37] Fischer Walker CL, Lamberti L, Adair L, Guerrant RL, Lescano AG, Martorell R, et al. Does childhood diarrhea influence cognition beyond the diarrhea-stunting pathway? PloS one. 2012;7(10):e47908. doi: 10.1371/journal.pone.0047908 23118906 PMC3485308

[38] Guerrant RL, DeBoer MD, Moore SR, Scharf RJ, Lima AA. The impoverished gut—a triple burden of diarrhoea, stunting and chronic disease. Nature reviews Gastroenterology & hepatology. 2013;10(4):220–9. doi: 10.1038/nrgastro.2012.239 23229327 PMC3617052

[39] Checkley W, Buckley G, Gilman RH, Assis AM, Guerrant RL, Morris SS, et al. Multi-country analysis of the effects of diarrhoea on childhood stunting. International journal of epidemiology. 2008;37(4):816–30. doi: 10.1093/ije/dyn099 18567626 PMC2734063

[40] Lutter C, Mora J, Habicht JP, Rasmussen K, Robson D, Sellers S, et al. Nutritional supplementation: effects on child stunting because of diarrhea. The American journal of clinical nutrition. 1989;50(1):1–8. doi: 10.1093/ajcn/50.1.1 2750681

